# New and little-known ant species (Hymenoptera, Formicidae) from Bulgaria

**DOI:** 10.3897/BDJ.10.e83658

**Published:** 2022-05-09

**Authors:** Albena Lapeva-Gjonova, Lech Borowiec

**Affiliations:** 1 Sofia University, Sofia, Bulgaria Sofia University Sofia Bulgaria; 2 University of Wroclaw, Wroclaw, Poland University of Wroclaw Wroclaw Poland

**Keywords:** ants, Bulgaria, distribution, Formicidae, new records

## Abstract

**Background:**

Many faunistic studies on the myrmecofauna of Bulgaria have been carried out and about 180 ant species (Hymenoptera, Formicidae) from 43 genera and six subfamilies have been discovered as a result. Although the Bulgarian ant fauna is considered to be relatively well studied, the finding of unrecorded species continues, especially amongst the rare social parasites and the species with a more southern distribution in the Balkans.

**New information:**

The current study presents data on 11 ant species recorded for the first time in Bulgaria (*Messorhellenius* Agosti & Collingwood, 1987, *M.mcarthuri* Steiner et al., 2018, *Crematogasterionia* Forel, 1911, *Monomoriummonomorium* Bolton, 1987, *Temnothoraxaeolius* (Forel, 1911), T.cf.exilis(formdarii Forel, 1911), *T.finzii* (Menozzi, 1925), *T.rogeri* Emery, 1869, Tetramoriumcf.punicum, *Plagiolepisxene* Stärcke, 1936 and *Lasiusreginae* Faber, 1967), as well as new locality data on 15 rarely found species. Some of the species, such as *Hypoponeraeduardi* (Forel, 1894), *Strumigenysargiola* (Emery, 1869), *Temnothoraxgraecus* (Forel, 1911), *Tetramoriumdiomedeum* Emery, 1908, *Camponotusionius* Emery, 1920 and *C.tergestinus* Müller, 1921, have been known so far only from a single locality in Bulgaria.

The dataset of all records presented in this work was published separately through Global Biodiversity Information Facility (GBIF, https://doi.org/10.15468/mngbzp).

## Introduction

Ants (family Formicidae) in Bulgaria, with about 180 reported species, represent one of the richest fauna on the Balkan Peninsula. This high number is due to the country’s heterogeneous topography, proximity to large water basins and the presence of sub-Mediterranean climatic influence in the southern regions. Only Greek myrmecofauna — with at least 315 known species — outnumbers the diversity reported from Bulgaria (Salata and Borowiec 2018)⁠.

The latest catalogue of the myrmecofauna of Bulgaria (Lapeva-Gjonova et al. 2010) was prepared, based on already published data on 163 ant species. Regardless, it significantly increased the knowledge on species diversity as the previously published review listed only 111 species (Atanassov and Dlussky 1992). Since the latest catalogue was published, 33 species have been added and important progress has been made in taxonomic studies focused on the diversity of cryptic species of the genera *Lasius*, *Messor*, *Temnothorax* and *Tetramorium* (Lapeva-Gjonova and Kiran 2012, Borowiec 2014, Csősz et al. 2014, Lapeva-Gjonova et al. 2014, Csősz et al. 2015, Seifert and Csősz 2015, Seifert 2016, Seifert and Galkowski 2016, Kiran et al. 2017, Wagner et al. 2017, Csősz et al. 2018, Steiner et al. 2018, Borowiec et al. 2019, Bračko et al. 2019, Ljubomirov 2019, Lapeva-Gjonova and Ljubomirov 2020, Seifert 2020, Lapeva-Gjonova and Radchenko 2021).

The present study adds new species records of taxa that were previously not known in Bulgaria, corrects some historical identifications and reports new localities of little-known species in the country. However, the doubtful presence of some species and the unidentified ant materials collected from some genera makes the final list incomplete.

## Materials and methods

The present study is based on ant materials collected during field trips to several sites, mainly in the southern regions of Bulgaria: the mountains — Strandzha, Sakar, the Eastern Rhodopes, Pirin, Belasitsa, Ograzhden and Maleshevska — as well as the Thracian Plane and Struma Valley in the period 1994-2021. However, the majority of new ant records were found in the Eastern Rhodopes. The main collection method was by hand, unless other methods, such as pitfall and tree traps, sifting, sweeping, light traps, Malaise traps, suction sampler, were noted. The specimens were deposited in the collection of the Faculty of Biology, Sofia University (BFUS), if not otherwise specified. The following abbreviations are used for ant castes in the results: q. - queen/s, m. - male/s, w. - worker/s; for collectors: ALG – A. Lapeva-Gjonova. The dataset of all records was published separately through GBIF (Lapeva-Gjonova and Borowiec 2022).

## Checklists

### Ponerinae

#### Hypoponera eduardi

(Forel, 1894)

162CE7BA-B56A-5C41-9263-B85DA1875A18

##### Distribution

New records: Maleshevska Mt., Dobri laki vill., 30.07.-20.08.2002, pitfall traps, 1 m., leg. T. Ljubomirov; Thracian Plane, Bodrovo vill., 03.03.2010, collected by suction sampler, 1 w., leg. ALG. Detailed occurrence data: Lapeva-Gjonova and Borowiec (2022).

##### Notes

This Mediterranean species was reported from Bulgaria just once in the region of Petrich (Atanassov and Dlussky 1992).

### Proceratiinae

#### Proceratium melinum

(Roger, 1860)

3D974BF4-60B8-5903-BF66-7A214AB8B39B

##### Distribution

New records: Belogradchik, 28.09.2009, 1 w., leg. P. Mitov; East Rhodopes, Kremen vill., 21.07.2009, 1 w., leg. ALG; Thracian Plane, Brestovitsa vill., 07.04.2012, sifting, 1 w., leg. R. Bekchiev. Detailed occurrence data: Lapeva-Gjonova and Borowiec (2022).

##### Notes

*Proceratiummelinum* is rarely collected due to its subterranean lifestyle and small colonies, although it is widespread in the Palaearctic. It is known in Bulgaria in a small number of localities (Dobrudzha, Thracian Lowland (Svilengrad), Struma Valley (Petrich, Sandanski) and Burgas) (Forel 1892, Atanassov and Dlussky 1992, Lapeva-Gjonova et al. 2010).

### Myrmicinae

#### Crematogaster ionia

Forel, 1911

CC22FC3F-7990-5ACE-8A72-638E3C13502F

##### Distribution

New records: Southern Black Sea coast, near Sinemorets vill., mouth Veleka River, 16.04.2009, sifting, 2 w., leg. R. Bekchiev; near Sinemorets vill., Butamyata loc., July 2010, pitfall traps, 1 w., leg. R. Kostova; East Rhodopes, Strazhets vill., 03.05.2009, 2 w., leg. ALG; Strandzha Mt., near Slivarovo vill, Shafaryitsa loc., waterside of Rezovska River, June 2010, pitfall traps, 1 w., leg. P. Mitov, R. Kostova, O. Sivilov. Detailed occurrence data: Lapeva-Gjonova and Borowiec (2022).

##### Notes

First record for Bulgaria. This species is known from the north-eastern Mediterranean Region.

#### Crematogaster gordani

Karaman, 2008

45206A6E-E9DA-53B2-95B5-23EE89E7AD22

##### Distribution

New records: Southern Black Sea coast: Chernomorets, 27.07.2006, 3 m., 7 w.; same place, 31.07.2006, 1 w., leg. and det. L. Borowiec (DBET); Sinemorets, Butamyata loc., 16.04.2009, 1 q., 4 w., leg. ALG. Detailed occurrence data: Lapeva-Gjonova and Borowiec (2022).

##### Notes

*Crematogastergordani* was described from Montenegro by Karaman (2008). Presented record from Chernomorets is the sample based on which the species was reported from Bulgaria (Borowiec 2014). The second locality also is situated on the southern Black Sea coast.

#### Crematogaster lorteti

Forel, 1910

4B0DC0B5-059E-5110-9853-67CAFDDE8489

##### Distribution

New records: East Rhodopes: Pastrook vill., sweeping, 2 w., leg. I. Gjonov; Strazhets vill., 05.09.2010, 4 w., leg. ALG; Kazak vill., 05.09.2010, 11 w., leg. ALG; Meden buk vill., 09.04.2013, 11 w., leg. ALG; Madzharovo, Gluhite kamani loc., 11.04.2013, 1 w., leg. ALG; Svirachi vill., 22.04.2014, 10 w., leg. ALG; Vetrushka vill., 01.06.2015, 1 w., leg. ALG; Sakar Mt., Mihalich vill., 03.05.2019, 20 w., leg. ALG; Struma Valley, Lebnitsa vill., 06.08.2019, light trap, 1 q., leg. ALG; Besapari hills, Isperihovo vill., 12.04.2021, 25 w., leg. ALG. Detailed occurrence data: Lapeva-Gjonova and Borowiec (2022).

##### Notes

This species is known from the north-eastern and eastern Mediterranean regions. The records of *Crematogasterauberti* Emery, 1869 from Struma Valley in Bulgaria (Lapeva-Gjonova 2011) should be assigned to *C.lorteti*.

#### Messor hellenius

Agosti & Collingwood, 1987

178B5C6B-3D99-5FD1-BF87-2F21ACEE1858

##### Distribution

**Figure 1.**
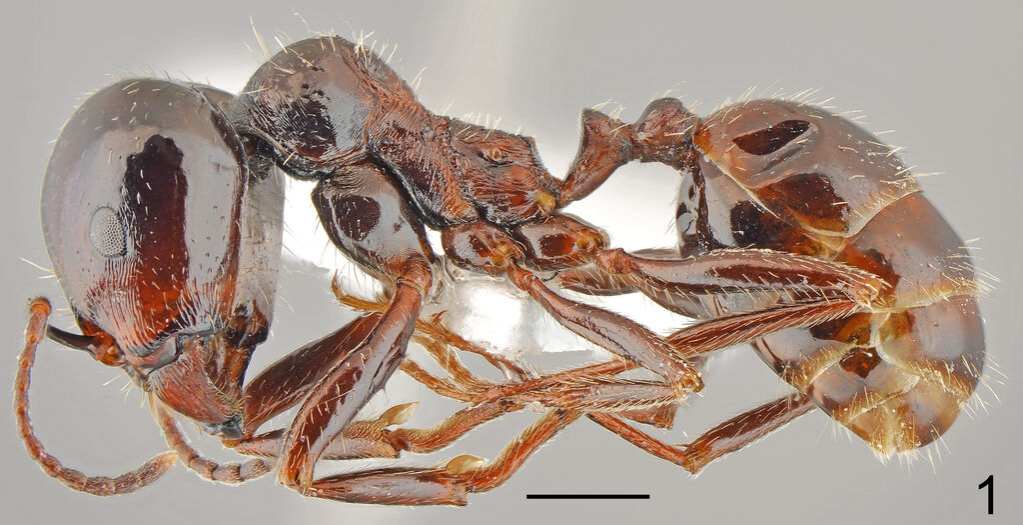
*Messorhellenius*, major lateral: dark specimen from Meden buk vill.; scale bar 1 mm.

**Figure 2.**
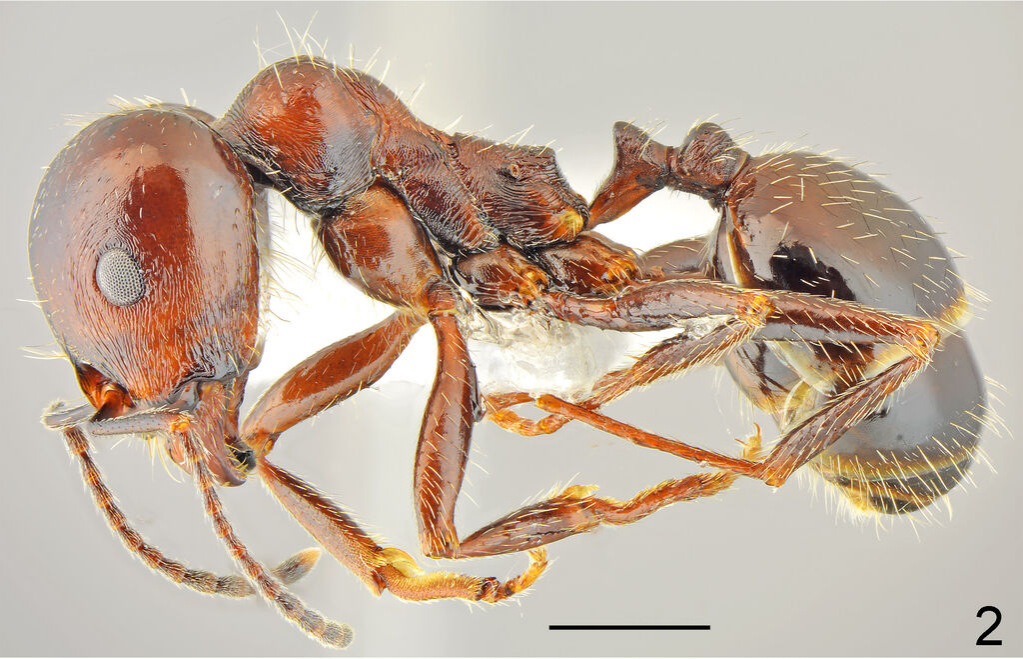
*Messorhellenius*, major lateral: pale specimen from Lebnitsa vill.; scale bar 1 mm.

New records: Ograzhden Mt.: Drenovo vill., 06.04.2010, 7 w., leg. ALG; Churilovo vill., 10.09.2021, 1 q., 3 w., leg. ALG; East Rhodopes: Meden buk vill., 22.02.2012, 3 w., leg. ALG (Fig. 1); Dolna Kula vill., 19.04.2012, 1 q., 3 w.; same place, 23.04.2021, 1 q., 15 w., leg. ALG; North Black Sea coast, Varna, Morska Gradina Park, 20.07.2017, 10 w., leg. ALG; Struma Valley, Lebnitsa vill., 06.08.2019, 13 w., leg. ALG (Fig. 2); Burgas District, Tranak vill., 01.01.2021, 5 w., leg. M. Mohamed; Pirin Mt., Stara Kresna vill., 11.05.2021, 12 w., leg. ALG; Southern Black Sea coast: Chernomorets, 20.07.2006, 5 w., leg. and det. L. Borowiec (DBET); Primorsko, 27.05.2011, 6 w., leg. ALG; Maslen nos cape, 04.06.2021, 20 w., leg. ALG. Detailed occurrence data: Lapeva-Gjonova and Borowiec (2022).

##### Notes

First record for Bulgaria. This species is known from Greece, the western coast of Anatolia and the European part of Turkey (Salata and Borowiec 2019a, Kiran and Karaman 2020). Borowiec and Salata (2018) suggested that the previous records of *M.capitatus* (Latreille, 1798) from eastern parts of the Balkan Peninsula concern *M.hellenius*. In this regard, it is very likely that the data on *M.capitatus* from the northern Black Sea coast (Markó and Csősz 2002) refer to *M.hellenius*.

#### Messor mcarthuri

Steiner et al., 2018

063185B5-1D4C-5F97-A071-417681804580

##### Distribution

**Figure 3.**
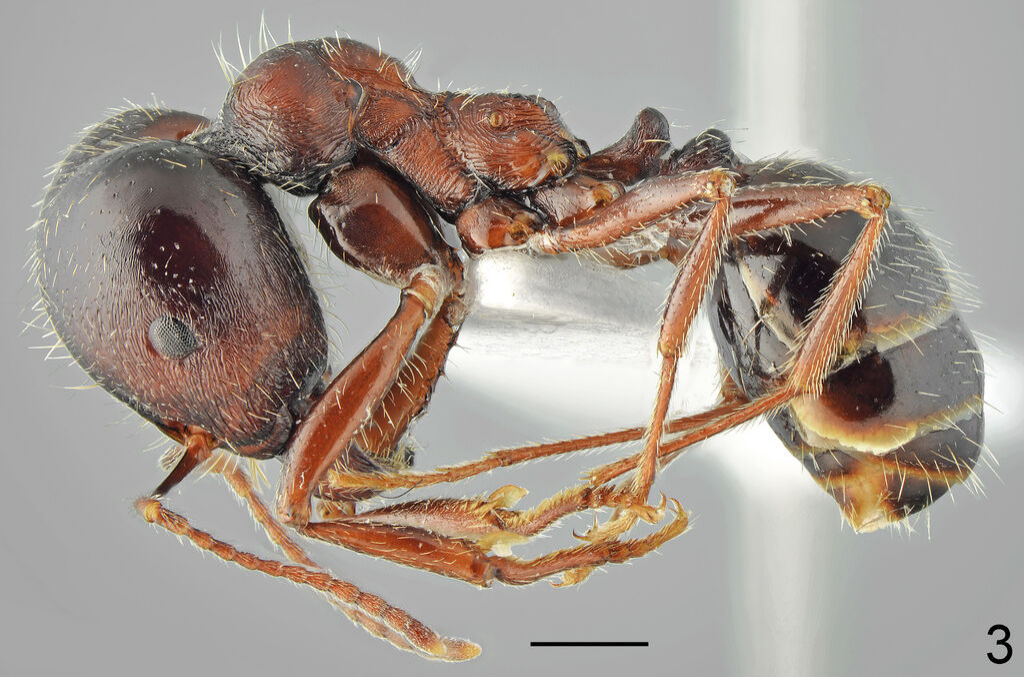
*Messormcarthuri*, major lateral: specimen from Rezovo vill.; scale bar 1 mm.

**Figure 4.**
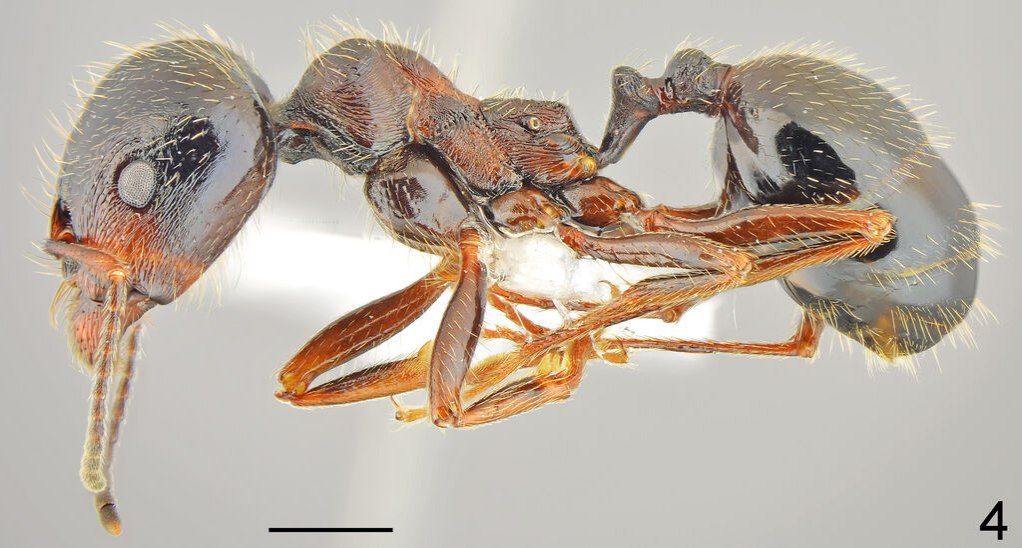
*Messormcarthuri*, major lateral: specimen from Golyam Dervent vill. with intermediate characters between *M.mcarthuri* and *M.hellenius*; scale bar 1 mm.

New records: Southern Black Sea coast, Rezovo vill., 09.05.2009, 1 w., leg. ALG (Fig. 3); Strandzha Mt., Malko Tarnovo, Propada loc., 27.05.2009, 1 w., leg. ALG; East Rhodopes: Zornitsa vill., 19.04.2012, 5 w., leg. ALG; Meden buk vill., 09.04.2013, 8 w., leg. ALG; Gorni Glavanak vill., 01.05.2016, 6 w., leg. ALG; Sakar Mt., Matochina vill., 03.05.2019, 2 w., leg. ALG; Dervent Heights, Golyam Dervent vill., 06.06.2021, 12 w., leg. ALG (Fig. 4). Detailed occurrence data: Lapeva-Gjonova and Borowiec (2022).

##### Notes

First record for Bulgaria. This is a recently described member of the *Messorstructor* species group (Steiner et al. 2018), distributed in Greece and Turkey (Kiran and Karaman 2020, Borowiec et al. 2021). Specimens from the vicinity of the Meden buk and Golyam Dervent villages have intermediate characteristics between *M.mcarthuri* and *M.hellenius*, which may indicate a possible hybridisation between these species (Fig. 4).

#### Monomorium monomorium

Bolton, 1987

6968E2B6-D3BA-5211-A389-3005FF0E3B8E

##### Distribution

**Figure 5.**
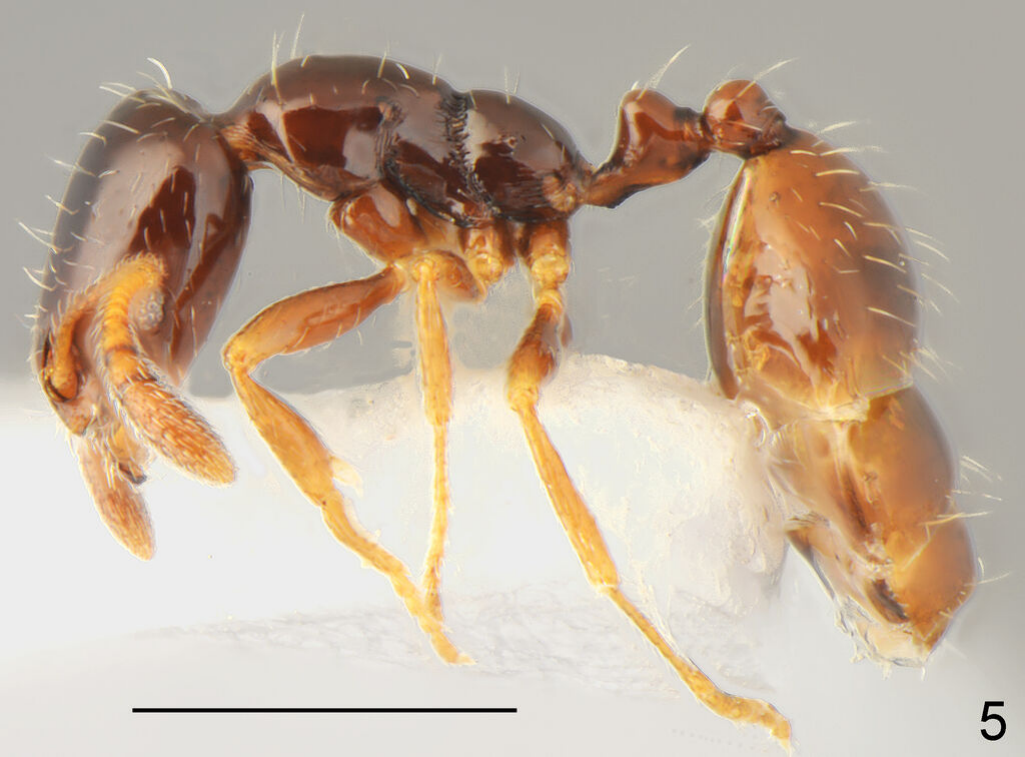
*Monomoriummonomorium*, worker lateral: specimen from Oreshino vill.; scale bar 0.5 mm.

New records: Sakar Mt., Matochina vill., 30.04.2011, 2 w.; same place, 03.05.2019, 2 w., leg. ALG; East Rhodopes: Oreshino vill., 21.04.2012, 5 w., leg. ALG (Fig. 5); Meden buk vill., 03.07.2014, 8 w., leg. ALG. All specimens were collected by a suction sampler. Detailed occurrence data: Lapeva-Gjonova and Borowiec (2022).

##### Notes

First record for Bulgaria. *Monomoriummonomorium* occurs in the Mediterranean Region and it is the only outdoor-living ant species of the *Monomorium* genus in Bulgaria.

#### Stenamma striatulum

Emery, 1895

DA7FCE2F-8303-504C-BC28-FF75FE2182B4

##### Distribution

New records: Zemen Gorge, Razhdavitsa vill. mouth of Shegava River, 09.08.2004, 4 w., leg. Y. Petrova; East Rhodopes, Kremen vill., 21.07.2009, 1 w., leg. ALG. Detailed occurrence data: Lapeva-Gjonova and Borowiec (2022).

##### Notes

This is a southern European species with a range extending to Anatolia. It was reported only from Strandzha Mountain in Bulgaria (Lapeva-Gjonova and Kiran 2012).

#### Strumigenys argiola

(Emery, 1869)

418C7C9A-A25A-5928-BB0A-CC90C55C43F9

##### Distribution

New records: Western Predbalkan, Banitsa vill., 21.08-28.08.1994, Malaise traps, 3 m., leg. T. Ljubomirov. Detailed occurrence data: Lapeva-Gjonova and Borowiec (2022).

##### Notes

Despite the rare finding of *Strumigenysargiola*, it is widespread in Europe and North Africa. This species was reported very recently for the first time from one locality in Bulgaria (Northeast Bulgaria, SW from Balchik) (Lapeva-Gjonova and Ljubomirov 2020)⁠. The presented record supplements the knowledge on its distribution in Bulgaria.

#### Temnothorax aeolius

(Forel, 1911)

CCD4CF8C-9D1A-538F-8702-BE19E776D116

##### Distribution

New records: South Pirin Mt., Kalimantsi vill., 06.04.-10.05.2002, pitfall traps, 1 w.; same place, 06.08-08.09.2002, pitfall traps, 1 w., leg. M. Langourov; Struma Valley, Kamenitsa vill., 23.06-08.08.2002, pitfall traps, 1 w., leg. D. Chobanov. Detailed occurrence data: Lapeva-Gjonova and Borowiec (2022).

##### Notes

First record for Bulgaria. A rarely collected ant that is a member of the *Temnothoraxgraecus* species group (Salata and Borowiec 2019b). This species is known from Turkey, its type locality, as well as from Israel and the Greek islands (Borowiec and Salata 2012). It was recently recorded in Greek Thrace (Bračko et al. 2016).

#### Temnothorax bulgaricus

(Forel, 1892)

89AD46E9-6094-5704-BF99-46C415F99A66

##### Distribution

New records: South Pirin Mt., Kalimantsi vill., 10.05.-01.06.2002, pitfall traps, 1 w., leg. M. Langourov; Struma Valley, Kamenitsa vill., 31.05-23.06.2002, tree traps, 1 w., leg. M. Langourov; East Rhodopes, Strazhets vill., 03.05.2009, 2 w., leg. ALG; Sakar Mt., Radovets vill., 30.04.2011, sweeping, 3 w., leg. I. Gjonov; East Rhodopes, Meden buk vill., 09.04.2013, 1 w., leg. ALG. Detailed occurrence data: Lapeva-Gjonova and Borowiec (2022).

##### Notes

After the description of *T.bulgaricus* from Bulgaria (Forel 1892), it has been reported from a number of places in the southern Balkans and Turkey. It was found in several thermophilous sites (Sliven, Zemen Gorge, Petrich, Asenovgrad, Obzor) in Bulgaria (Atanassov and Dlussky 1992). Here we provide additional findings as South Pirin Mt., East Rhodopes Mt. and Sakar Mt. are hitherto unknown distribution regions.

#### Temnothorax cf. exilis

(form darii Forel, 1911)

9460942B-EDB9-5900-A5B2-184D03AE7BD3

##### Distribution

New records: South Pirin Mt., Kalimantsi vill., 06.04-10.05.2002, pitfall traps, 2 w., leg. M. Langourov; East Rhodopes, Svirachi vill., 22.04.2014, 1 w., leg. ALG. Detailed occurrence data: Lapeva-Gjonova and Borowiec (2022).

##### Notes

First record for Bulgaria. This is a common Balkan form of the species belonging to the *Temnothoraxexilis* group. It was described from the vicinity of Izmir in Turkey as var. darii (Forel 1911) and synonymised with *T.exilis* by Baroni Urbani (1971). It is likely that the Balkan population is not conspecific with true *T.exilis*, described from the vicinity of Naples in Italy. Until the situation is clarified, we will leave the name of the morphospecies as Temnothoraxcf.exilis(formdarii Forel, 1911).

#### Temnothorax finzii

(Menozzi, 1925)

908535DF-6C0A-52E4-AD5F-798DB5C11921

##### Distribution

**Figure 6.**
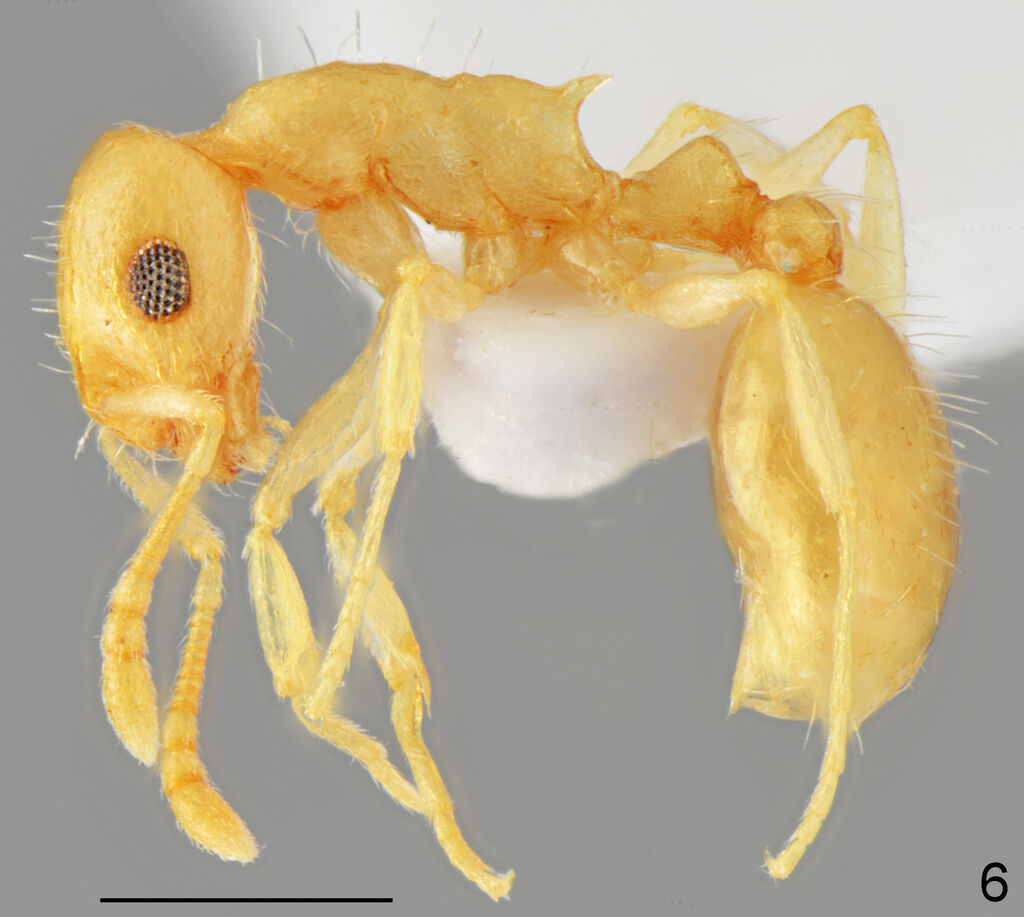
*Temnothoraxfinzii*, worker lateral: specimen from Kalimantsi vill.; scale bar 0.5 mm.

New record: South Pirin Mt., Kalimantsi vill., 06.08-08.09.2002, pitfall traps, 1 w., leg. M. Langourov (Fig. 6). Detailed occurrence data: Lapeva-Gjonova and Borowiec (2022).

##### Notes

First record for Bulgaria. Extremely rarely reported species known from Italy and the Republic of North Macedonia (Bračko et al. 2014), as its presence in Anatolia is doubtful (Kiran and Karaman 2020).

#### Temnothorax graecus

(Forel, 1911)

E84EA058-2CE2-529B-9892-A13FAC1A4C55

##### Distribution

New record: South Pirin Mt., Kalimantsi vill., 06.04-10.05.2002, pitfall traps, 1 w., leg. M. Langourov; Struma Valley, Kamenitsa vill., 31.05-23.06.2002, tree traps, 3 w., leg. M. Langourov. Detailed occurrence data: Lapeva-Gjonova and Borowiec (2022).

##### Notes

*Temnothoraxgraecus* has a range restricted to Greece, the Republic of North Macedonia and Bulgaria. Prior to this study, there was only one record of this species from Bulgaria (Central Stara Planina Mts: Gabrovo) (Lapeva-Gjonova et al. 2010).

#### Temnothorax rogeri

Emery, 1869

4696CABA-B05C-574E-A5F2-5A86C2F31358

##### Distribution

New record: South Pirin Mt., Kalimantsi vill., 10.05.-01.06.2002, 01-22.06.2002, 06.08-08.09.2002, pitfall traps, 8 w., leg. M. Langourov. Detailed occurrence data: Lapeva-Gjonova and Borowiec (2022).

##### Notes

First record for Bulgaria. *Temnothoraxrogeri* is an eastern Mediterranean species, known from Croatia, Greece, Montenegro and Turkish Thrace (Borowiec and Salata 2018, Kiran and Karaman 2020). Recently, it was recorded in Slovakia, but most likely this record is based on an introduced specimen (Klesniaková et al. 2018). Morphologically, it is very close to *T.recedens* (Nylander, 1856), which is known from the southern regions and the Black Sea coast in Bulgaria (Atanassov and Dlussky 1992, Lapeva-Gjonova et al. 2010) .

#### Tetramorium diomedeum

Emery, 1908

219C141A-622F-522E-B800-3C0017F00044

##### Distribution

New records: Struma Valley, Kamenitsa vill., tree traps, 31.05-23.06.2002, 2 w., leg. M. Langourov; East Rhodopes, Meden buk vill., 04.05.2009, 2 q., leg. ALG; South Black Sea coast: Silistar, 28.04.2011, 10 w., leg. ALG; Primorsko, Maslen Nos cape, 25.06.2014, 7 q., 7 m., 10 w., leg. ALG. Detailed occurrence data: Lapeva-Gjonova and Borowiec (2022).

##### Notes

*Tetramoriumdiomedeum* has east Mediterranean distribution—from Italy to the Anatolian part of Turkey (Salata et al. 2020). Prior to this study, *T.diomedeum* was reported from Bulgaria just once from Ahtopol (Southern Black Sea coast) (Csősz and Schulz 2010).

#### Tetramorium cf. punicum

53A09EF8-D5AF-59DE-8D92-3043293933E1

##### Distribution

New record: East Rhodopes, Meden buk vill., 22.04.2021, 12 w., leg. ALG. Detailed occurrence data: Lapeva-Gjonova and Borowiec (2022).

##### Notes

At least three morphospecies close to *Tetramoriumpunicum* (Smith, 1861), described from Israel, have been distinguished in the southern Balkans and the Greek islands. Accurate identification of this species complex requires the study of sexual castes. As our material consists only of workers, the species-level determination is not possible.

### Formicinae

#### Camponotus ionius

Emery, 1920

34918EBC-37CE-5BCF-9F51-45DE57C05B9E

##### Distribution

New record: Struma Valley, Kamenitsa vill., 31.05.-03.06.2002, pitfall traps, 3 w., leg. M. Langourov. Detailed occurrence data: Lapeva-Gjonova and Borowiec (2022).

##### Notes

The only published record comes from the hill of Kozhuh, situated not far from the new locality. As noted by Borowiec and Salata (2018), this species is widespread in the Balkans and Turkey.

#### Camponotus oertzeni

Forel, 1889

F6340C1F-70CD-5CD5-AE45-94BFBD3732BC

##### Distribution

North Black Sea coast, Cape Kaliakra, 23.06.2008, 6 w., leg. ALG; East Rhodopes, Strazhets vill., 03.05.2009, 2 w., leg. ALG; Gaberovo vill., 10.04.2013, 7 w., leg. ALG; Oreshari vill., 22.04.2014, 15 w., leg. ALG; Meden buk vill., 22.04.2021, 10 w., leg. ALG; Maleshevska Mt., Gorna Breznitsa vill., 27.03.2012, 1 q., 10 w., leg. ALG; Pirin Mt., Vlahi vill., 27.03.2012, 15 w., leg. ALG; Stara Kresna vill., 06.05.2013, 20 w., leg. ALG; Slavyanka Mt., Goleshovo vill., 04.05.2013, 7 w., leg. ALG. Detailed occurrence data: Lapeva-Gjonova and Borowiec (2022).

##### Notes

*Camponotusoertzeni* is known from Greece, Serbia, Iran and Turkey (Borowiec and Salata 2018) and its first published record from Bulgaria was given under the name *C.pilicornis* (Roger, 1859) in Lapeva-Gjonova and Santamaria (2011).

#### Camponotus tergestinus

Müller, 1921

95C34684-BE38-5C92-AB83-E0E6EC6CCED7

##### Distribution

New record: Strandzha Mt., Bliznak vill., Bataka loc., July 2010, pitfall traps, 1 w., leg. R. Kostova. Detailed occurrence data: Lapeva-Gjonova and Borowiec (2022).

##### Notes

*Camponotustergestinus* is a rare arboricolous ant species, nesting in oaks. It has scattered east Mediterranean distribution—from Italy to the Anatolian part of Turkey (Markó et al. 2009, Bračko 2017). In Bulgaria, it was known from only one locality on the southern Black Sea coast (an oak forest near the village of Sinemorets) (Lapeva-Gjonova and Kiran 2012).

#### Camponotus aegaeus

Emery, 1915

BD1D6423-3633-5451-BE31-F8191970374D

##### Distribution

New records: East Rhodopes, Meden buk vill., 03.07.2014, 1 w.; same place, 22.04.2021, 2 w., leg. ALG; Ograzhden Mt., Drakata vill., 09.09.2021, 10 w., leg. ALG. Detailed occurrence data: Lapeva-Gjonova and Borowiec (2022).

##### Notes

This is a species from the *Camponotuskiesenwetteri* group, which occurs in Greece, Turkey, the Republic of North Macedonia and Bulgaria (Salata et al. 2019). In Bulgaria, the species was reported in two localities: Struma Valley and South Pirin Mt. (Lapeva-Gjonova 2011).

#### Cataglyphis viaticoides

(André, 1881)

1A9D2B35-057C-5D15-8AA2-18EA0F08ABC1

##### Distribution

**Figure 7.**
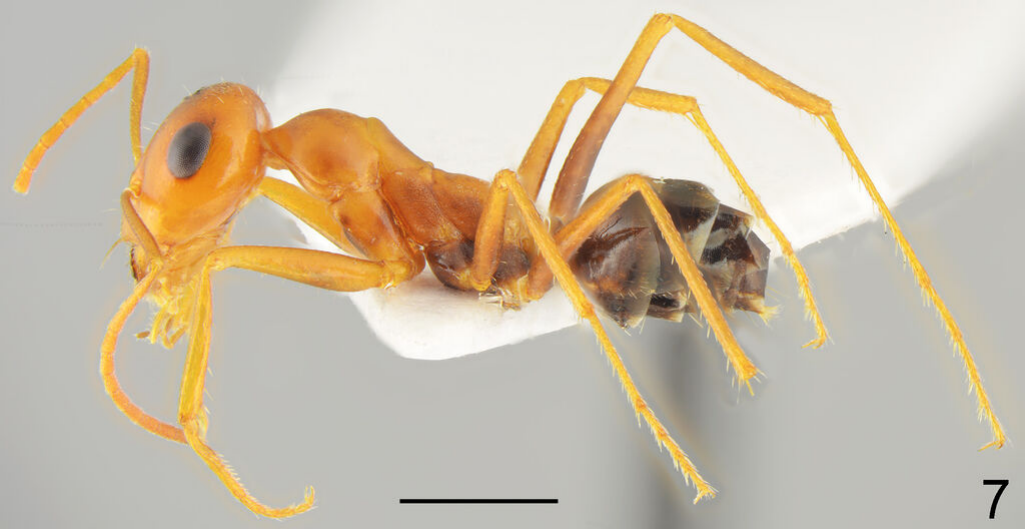
*Cataglyphisviaticoides*, worker lateral: specimen from Svirachi vill.; scale bar 1 mm.

New record: East Rhodopes, Svirachi vill., 02.06.2015, 6 w., leg. ALG (Fig. 7). Detailed occurrence data: Lapeva-Gjonova and Borowiec (2022).

##### Notes

In the last revision of *Cataglyphislivida* complex, Salata et al. (2021) proposed *Cataglyphisviaticoides* as a senior synonym of *Cataglyphislividabulgarica* Atanassov, 1982. It is known from few sites in East Rhodopes in Bulgaria (Atanassov and Dlussky 1992) and its general distribution covers the south-eastern Balkans and Asia Minor (Salata et al. 2021).

#### Formica clara

Forel, 1886

E2868E2C-37E5-5EEF-8989-2CE64370337C

##### Distribution

New records: East Rhodopes, Meden buk vill., 04.05.2009, 7 w.; same place, 22.04.2021, 3 w., leg. ALG; Southern Black Sea coast, Primorsko, Perla loc., 03.06.2021, 5 w., leg. ALG. Detailed occurrence data: Lapeva-Gjonova and Borowiec (2022).

##### Notes

Although this species has a wide range of distribution (from Europe and Anatolia to the Near East), it is limited to xerothermous grasslands at relatively low altitudes (Borowiec and Salata 2018, Seifert 2018). Only three localities are known so far for *Formicaclara* in Bulgaria (Lapeva-Gjonova et al. 2010) and the Eastern Rhodopes is a new distribution area.

#### Lasius reginae

Faber, 1967

50600C4A-E38B-5261-9648-F3028DEF1DD6

##### Distribution

**Figure 8.**
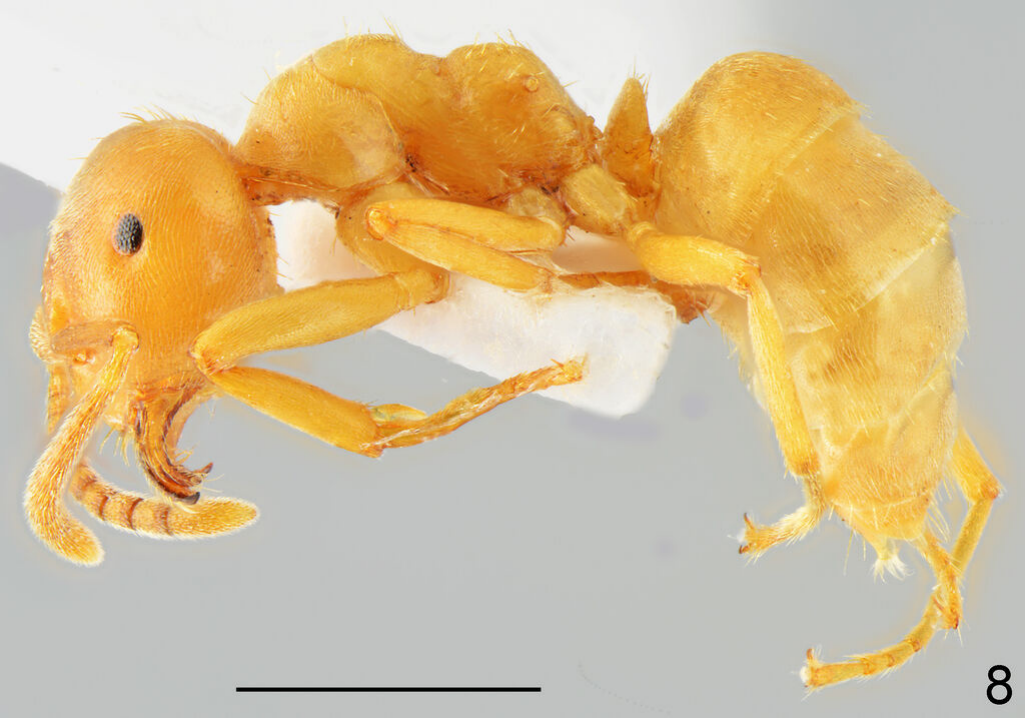
*Lasiusreginae*, worker lateral: specimen from Goleshovo vill.; scale bar 1 mm.

New record: Slavyanka Mt., Goleshovo vill., 16.08.2014, 20 w. (Fig. 8) in a nest of *Lasiusalienus* (Förster, 1850) located at the side of a path along the edge of a forest. Detailed occurrence data: Lapeva-Gjonova and Borowiec (2022).

##### Notes

First record for Bulgaria. This is a very rarely recorded temporary social parasite of *Lasiusalienus* recorded from several European countries, as well as from Turkey and Mongolia (Aibek and Yamane 2010, Borowiec 2014). *Lasiusreginae* joins *L.carniolicus* Mayr, 1861 as the second member of the subgenusAustrolasius known from Bulgaria. It differs from the latter in a reduced chaetotaxy (Fig. 8).

#### Plagiolepis xene

Stärcke, 1936

4F5D0F4B-5AAA-5FDB-B53C-C62F3F57CD39

##### Distribution

New record: East Rhodopes, Bryagovets vill., 06.04.2013, 7 q. in a nest of *Plagiolepispygmaea* (Latreille, 1798), leg. ALG; Ograzhden Mt., Drakata vill., 09.09.2021, 6 q. in a nest of *Pl.pygmaea*, leg. ALG. Detailed occurrence data: Lapeva-Gjonova and Borowiec (2022).

##### Notes

First record for Bulgaria. *Plagiolepisxene* is a rare workerless inquiline in nests of *Plagiolepispygmaea*. Its distribution range covers southern and central Europe to Anatolia.

## Discussion

In the present study, 11 new species of ants are reported for the fauna of Bulgaria — *Messorhellenius*, *M.mcarthuri*, *Crematogasterionia*, *Monomoriummonomorium*, *Temnothoraxaeolius*, *T* . cf.exilis form (darii) , *T.finzii*, *T.rogeri*, Tetramoriumcf.punicum, *Plagiolepisxene* and *Lasiusreginae*. These new discoveries are added to the already known about 180 species. The number of ant species in Bulgaria is far higher than in the other Balkan countries, except Greece — most likely due to high number of endemic species known from this country.

Quite a few ant species in Bulgaria remain poorly studied due to their relatively rare detection, limited distribution or difficulties in identification. In the present study, for the first time, exact localities of *Crematogastergordani*, previously reported for Bulgaria by Borowiec (2014), are given. New localities are added for another 14 rarely registered species. Some of the species, such as *Hypoponeraeduardi*, *Strumigenysargiola*, *Temnothoraxgraecus*, *Tetramoriumdiomedeum*, *Camponotustergestinus* and *C.ionius*, have been known so far only from a single locality in Bulgaria and *Stenammastriatulum* from two localities.

Almost all species included in this study, with the exception of *Lasiusreginae*, are characteristic elements of southern European or eastern Mediterranean faunal complexes. This is directly related to the established localities, mainly in the southern regions of the country and the Black Sea coast, where the impact of the Mediterranean climate is more noticeable.

The two rare members of the socially parasitic species, *Lasiusreginae* and *Plagiolepisxene*, were found together with their typical hosts: *Lasiusalienus* and *Plagiolepispygmaea*, respectively. Although their hosts can be common, both parasitic species are extremely rare, with *Lasiusreginae* listed as vulnerable (IUCN 2022).

It is expected that future taxonomic revisions of some problematic groups of species from the southern Balkans, for example, of the genera *Aphaenogaster*, *Messor*, *Temnothorax* and *Tetramorium*, will significantly clarify the boundaries between species and will increase the knowledge of the extremely diverse fauna in this region.

## Supplementary Material

XML Treatment for Hypoponera eduardi

XML Treatment for Proceratium melinum

XML Treatment for Crematogaster ionia

XML Treatment for Crematogaster gordani

XML Treatment for Crematogaster lorteti

XML Treatment for Messor hellenius

XML Treatment for Messor mcarthuri

XML Treatment for Monomorium monomorium

XML Treatment for Stenamma striatulum

XML Treatment for Strumigenys argiola

XML Treatment for Temnothorax aeolius

XML Treatment for Temnothorax bulgaricus

XML Treatment for Temnothorax cf. exilis

XML Treatment for Temnothorax finzii

XML Treatment for Temnothorax graecus

XML Treatment for Temnothorax rogeri

XML Treatment for Tetramorium diomedeum

XML Treatment for Tetramorium cf. punicum

XML Treatment for Camponotus ionius

XML Treatment for Camponotus oertzeni

XML Treatment for Camponotus tergestinus

XML Treatment for Camponotus aegaeus

XML Treatment for Cataglyphis viaticoides

XML Treatment for Formica clara

XML Treatment for Lasius reginae

XML Treatment for Plagiolepis xene

## References

[B7725579] Aibek U., Yamane S. (2010). Discovery of the subgenera *Austrolasius* and *Dendrolasius* of the ant genus *Lasius* (Hymenoptera, Formicidae) from Mongolia. Japanese Journal of Systematic Entomology.

[B7725616] Atanassov N., Dlussky G. M. (1992). Fauna Bulgarica. 22. Hymenoptera, Formicidae.

[B7725624] Baroni Urbani C. (1971). Studien zur Ameisenfauna Italiens XI. Die Ameisen des Toscanischen Archipels. Betrachtungen zur Herkunft der Inselfaunen. Revue Suisse de Zoologie.

[B7725651] Borowiec L., Salata S. (2012). Ants of Greece - checklist, comments and new faunistic data (Hymenoptera: Formicidae). Genus.

[B7725633] Borowiec L. (2014). Catalogue of ants of Europe, the Mediterranean Basin and adjacent regions (Hymenoptera: Formicidae). Genus.

[B7725669] Borowiec L., Salata S. (2018). New records of ants (Hymenoptera: Formicidae) from Epirus, Greece. Acta Entomologica Silesiana.

[B7725642] Borowiec L., Lapeva-Gjonova A., Salata S. (2019). Three species of *Aphaenogaster* Mayr, 1853 (Hymenoptera: Formicidae) new to the Bulgarian fauna. Acta Zoologica Bulgarica.

[B7725678] Borowiec L., Wieczorek K., Salata S. (2021). Review of ants (Hymenoptera: Formicidae) of the Dodecanese Archipelago, Greece. Annals of the Upper Silesian Museum in Bytom Entomology.

[B7725718] Bračko G., Wagner H. C., Schulz A., Gioahin E., Matičič J., Tratnik A. (2014). New investigation and a revised checklist of the ants (Hymenoptera: Formicidae) of the Republic of Macedonia. North-Western Journal of Zoology.

[B7725696] Bračko G., Kiran K., Karaman C., Salata S., Borowiec L. (2016). Survey of the ants (Hymenoptera: Formicidae) of the Greek Thrace. Biodiversity Data Journal.

[B7725687] Bračko G. (2017). First discoveries of colonies of the rare ant species *Camponotustergestinus* Müller, 1921 (Hymenoptera: Formicidae) in situ. Natura Sloveniae.

[B7725707] Bračko G., Lapeva-Gjonova A., Borowiec L., Polak S. (2019). *Aphaenogasterillyrica*, a new species from the mountains of the Balkan Peninsula. ZooKeys.

[B7725748] Csősz S., Schulz A. (2010). A taxonomic review of the Palaearctic *Tetramoriumferox* species-complex (Hymenoptera, Formicidae). Zootaxa.

[B7725757] Csősz S., Seifert B., Müller B., Trindl A., Trindl A., Schulz A., Heinze J. (2014). Cryptic diversity in the Mediterranean *Temnothoraxlichtensteini* species complex (Hymenoptera: Formicidae). Organisms Diversity and Evolution.

[B7725730] Csősz S., Heinze J., Mikó I. (2015). Taxonomic synopsis of the Ponto-Mediterranean ants of *Temnothoraxnylanderi* species-group. PLOS One.

[B7725739] Csősz S., Salata S., Borowiec L. (2018). Three Turano-European species of the *Temnothoraxinterruptus* group (Hymenoptera: Formicidae) demonstrated by quantitative morphology. Myrmecological News.

[B7727072] Forel A. (1892). Die Ameisenfauna Bulgariens. (Nebst biologischen Beobachtungen). Verhandlungen der Zoologisch-Botanischen Gesellschaft in Wien.

[B7725769] Forel A. (1911). Fourmis nouvelles ou intéressantes. Bulletin de la Société Vaudoise des Sciences Naturelles.

[B7725778] IUCN The IUCN Red List of Threatened Species. Version 2021-3.. https://www.iucnredlist.org.

[B7725786] Karaman M. (2008). Two new species of the *Crematogasterscutellaris* group, *Crematogastergordani*, sp. nov. and *C.montenigrinus* sp. nov. (Insecta: Hymenoptera: Formicidae) from Crna Gora (Montenegro) with key of this group from Southern Europe. Natura Montenegrina, Podgorica.

[B7725804] Kiran K., Karaman C., Aksoy V., Lapeva-Gjonova A. (2017). Two new species of the “ultimate” parasitic ant genus *Teleutomyrmex* Kutter, 1950 (Hymenoptera: Formicidae) from the Western Palaearctic. Myrmecological News.

[B7725795] Kiran K., Karaman C. (2020). Additions to the ant fauna of Turkey (Hymenoptera, Formicidae). Zoosystema.

[B7725813] Klesniaková M., Pavlíková A., Holecová M. (2018). *Temnothoraxrogeri* (Emery, 1869) becoming an established neozoon in Central Europe? (Hymenoptera, Formicidae). Spixiana.

[B7725831] Lapeva-Gjonova A., Antonova V., Radchenko A. G., Atanasova M. (2010). Catalogue of the ants (Hymenoptera: Formicidae) of Bulgaria. ZooKeys.

[B7725822] Lapeva-Gjonova A. (2011). First records of three ant species (Hymenoptera: Formicidae) from Bulgaria. Myrmecological News.

[B7725876] Lapeva-Gjonova A., Santamaria S. (2011). First records of Laboulbeniales (Ascomycota) on ants (Hymenoptera: Formicidae) in Bulgaria. ZooNotes.

[B7725840] Lapeva-Gjonova A., Kiran K. (2012). Ant fauna (Hymenoptera, Formicidae) of Strandzha Mountain (Istranca) and adjacent Black Sea coast. North-Western Journal of Zoology.

[B7725849] Lapeva-Gjonova A., Kiran K., Karaman C. (2014). First records of *Temnothoraxflavicornis* (Emery, 1870) (Hymenoptera: Formicidae) in Bulgaria and Turkey. Acta Zoologica Bulgarica.

[B7725858] Lapeva-Gjonova A., Ljubomirov T. (2020). First records of two *Strumigenys* ant species (Hymenoptera, Formicidae) from Bulgaria. Sociobiology.

[B7725867] Lapeva-Gjonova A., Radchenko A. G. (2021). Ant genus *Strongylognathus* (Hymenoptera, Formicidae) in Bulgaria: a preliminary review. Biodiversity Data Journal.

[B7741022] Lapeva-Gjonova A., Borowiec L. (2022). New and little-known ant species (Hymenoptera, Formicidae) from Bulgaria. Biodiversity Data Journal. Occurrence dataset.

[B7725894] Ljubomirov T., Bechev D., Georgiev D. (2019). Faunistic diversity of Vrachanski Balkan Nature Park. Part 2.

[B7725916] Markó B., Csősz S. (2002). Die europäischen Ameisenarten (Hymenoptera: Formicidae) des Hermannstädter (Sibiu, Rumänien) Naturkundemuseums I.: Unterfamilien Ponerinae, Myrmicinae und Dolichoderinae. Annales Historico-Naturales Musei Nationalis Hungarici.

[B7725925] Markó B., Ionescu-Hirsch A., Szász-Len A. (2009). Genus *Camponotus* Mayr, 1861 (Hymenoptera: Formicidae) in Romania: distribution and identification key to the worker caste. Entomologica Romanica.

[B7725934] Salata S., Borowiec L. (2018). Taxonomic and faunistic notes on Greek ants (Hymenoptera: Formicidae). Annals of the Upper Silesian Museum in Bytom, Entomology.

[B7725943] Salata S., Borowiec L. (2019). Preliminary contributions toward a revision of Greek *Messor* Forel, 1890 (Hymenoptera: Formicidae). Turkish Journal of Zoology.

[B7725952] Salata S., Borowiec L. (2019). Preliminary division of not socially parasitic Greek *Temnothorax* Mayr, 1861 (Hymenoptera, Formicidae) with a description of three new species. ZooKeys.

[B7725970] Salata S., Loss A. C., Karaman C., Kiran K., Borowiec L. (2019). Review of the *Camponotuskiesenwetteri* group (Hymenoptera, Formicidae) in the Aegean with the description of a new species. ZooKeys.

[B7828339] Salata Sebastian, Borowiec Lech, Trichas Apostolos (2020). Review of ants (Hymenoptera: Formicidae) of Crete, with keys to species determination and zoogeographical remarks. Monographs of the Upper Silesian Museum.

[B7725961] Salata S., Kiyani H., Minaei K., Borowiec L. (2021). Taxonomic review of the *Cataglyphislivida* complex (Hymenoptera, Formicidae), with a description of a new species from Iran. ZooKeys.

[B7726006] Seifert B., Csősz S. (2015). *Temnothoraxcrasecundus* sp. n. - a cryptic Eurocaucasian ant species (Hymenoptera, Formicidae) discovered by Nest Centroid Clustering. ZooKeys.

[B7725980] Seifert B. (2016). Inconvenient hyperdiversity - the traditional concept of “*Pheidolepallidula*” includes four cryptic species (Hymenoptera: Formicidae). Soil Organisms.

[B7726015] Seifert B., Galkowski C. (2016). The Westpalaearctic *Lasiusparalienus* complex (Hymenoptera: Formicidae) contains three species. Zootaxa.

[B7725989] Seifert B. (2018). The Ants of the Central and North Europe.

[B7725997] Seifert B. (2020). A taxonomic revision of the Palaearctic members of the subgenusLasius s.str (Hymenoptera, Formicidae). Soil Organisms.

[B7726040] Steiner F. M., Csősz S., Markó B., Gamisch A., Rinnhofer L., Folterbauer C., Hammerle S., Stauffer C., Arthofer W., Schlick-Steiner B. C. (2018). Turning one into five: Integrative taxonomy uncovers complex evolution of cryptic species in the harvester ant *Messor* "*structor*". Molecular Phylogenetics and Evolution.

[B7726060] Wagner H. C., Arthofer W., Seifert B., Muster C., Steiner F. M., Schlick-Steiner B. C. (2017). Light at the end of the tunnel: Integrative taxonomy delimits cryptic species in the *Tetramoriumcaespitum* complex (Hymenoptera: Formicidae). Myrmecological News.

